# The impact of an alkasite restorative material on the pH of *Streptococcus mutans* biofilm and dentin remineralization: an in vitro study

**DOI:** 10.1186/s12903-022-02354-4

**Published:** 2022-08-08

**Authors:** Pawinee Wiriyasatiankun, Rangsima Sakoolnamarka, Panida Thanyasrisung

**Affiliations:** 1 Department of Operative Dentistry, Faculty of Dentistry, Chulalongkorn University, Bangkok, 10330 Thailand; 2 Department of Microbiology and Center of Excellence on Oral Microbiology and Immunology, Faculty of Dentistry, Chulalongkorn University, Bangkok, 10330 Thailand

**Keywords:** Alkasite, Biofilm, Ion release, *Streptococcus mutans*, Hardness

## Abstract

**Background:**

It has been claimed that an alkasite restorative material can neutralize acids produced by cariogenic bacteria from released hydrogen ions and enable to remineralization via calcium and fluoride ions. However, there is no evidence to support this assertion. Therefore, the aims of this study were to investigate the effect of the alkasite restorative material on the pH of *Streptococcus mutans* biofilm and dentin hardness.

**Methods:**

*Streptococcus mutans* biofilms were formed on Filtek™ Z350 (FZ, a resin composite) and Cention® N (CN, the alkasite restorative material) and their pH determined after 24 h. Hydroxide, fluoride, and calcium-ions released from the materials were determined at 6 h, 1, 3, 7, 14, and 28 days. Dentin specimens were prepared from 14 human molars and divided into four quadrants. Quadrant 1 was a sound dentin control, quadrants 2–4 were chemically demineralized, and a cylinder of FZ and CN placed on the surfaces of quadrants 2 and 4, respectively. The microhardness of quadrants 1 and 3 were measured at depths of 20, 40, and 60 µm from the occlusal surface, and similarly of quadrants 2 and 4, after 30 days. Independent t-test, Mann–Whitney-U, and repeated-measure-ANOVA were used for data analysis.

**Results:**

The pH of biofilm on CN (4.45) was significantly higher (*p* < 0.05) than that on FZ (4.06). The quantity of all ions released from CN was significantly higher than from FZ. The hardness of demineralized dentin under CN was significantly higher than that of demineralized dentin at all depths, and higher than that of demineralized dentin under FZ at 20 and 40 µm.

**Conclusions:**

CN released hydroxide, fluoride, and calcium ions, which was associated with raising the biofilm pH and the hardness of demineralized dentin. All results indicated that CN had the potential to reduce the incidence of secondary caries.

## Background

Dental caries is one of the most common oral diseases related to dental biofilm [1], and *Streptococcus mutans* (*S. mutans*) is one of the main biofilm bacteria which produce acids from fermentable sugar and cause demineralization of the tooth surface [2, 3]. The ecological plaque hypothesis is widely recognized as the most plausible explanation for caries development. More than 700 bacterial species live in the oral cavity, which in health live in symbiosis. However, frequent sugar consumption can enhance biofilm acidogenicity (lower the pH) and promote a shift in the balance towards pathogenic bacteria, including *S. mutans*, which can survive in acidic conditions (the property of aciduricity). Prolonged low pH results in tooth demineralization and eventually, a frank cavity [4]. To control the disease of dental caries, neutralization of the acid may help in the maintenance of microbial symbiosis inside the biofilm [5].

An alkasite restorative material is a new category of filling material which is classified as a subgroup of resin composite [6]. The manufacturer (Ivoclar Vivadent, Schaan, Liechtenstein) claims that the main advantage of an alkasite material is that it can release hydroxide, calcium, and fluoride ions from its alkaline (calcium fluoro-silicate glass) filler [6]. Hydroxide ions present on the surface of the material may play an important role in neutralizing acids produced by cariogenic bacteria [7]. Furthermore, the release of calcium and fluoride ions from the filler is postulated to contribute to remineralization and the prevention of dental caries [6]. Calcium is necessary for remineralization, and fluoride plays an important role in accelerating the process [8].

There has not been any previous research on the influence of hydroxide ions produced from the alkasite material on the pH of biofilm, and only one study (using polarized light microscopy) has assessed the capacity of calcium and fluoride ions released by the alkasite material to inhibit demineralization [9]. Therefore, the aims of this laboratory study were to assess the potential of the alkasite material to: (a) raise the pH of a biofilm containing *S. mutans*; (b) remineralize demineralized dentin.

## Materials and methods

### Determination of biofilm pH and ion release

#### Material preparation

The sample size was calculated based on a previous study [7] using two independent means and a power of 90%, with significance level at 5%. Thirty-one specimens of each of two restorative materials: (1) alkasite (Cention® N; Ivoclar Vivadent**,** Schaan, Liechtenstein (CN)) and (2) resin composite (Filtek™ Z350XT; 3M ESPE, St. Paul, MN (FZ)) were prepared (Table 1). For CN, the powder bottle was shaken well, the scoop provided overfilled with powder and the excess powder scraped off. The liquid bottle was held vertically, and one drop of liquid extruded, ensuring that it was free of bubbles. One scoop of powder and one drop of liquid were mixed for 45–60 s. The mixed CN and the FZ were placed in acrylic molds, 10 mm diameter × 2 mm deep [10], the molds placed on a glass slide and covered with a second glass slide to extrude excess material. The discs of CN and FZ were cured for 40 s from the upper surface using a curing light (Elipar™; 3M ESPE, St. Paul, MN) with a light intensity $\ge$ 500 mW/cm^2^.

**Table 1:** Restorative materials

Material types	Compositions
*Alkasite: Cention® N (CN)* (Ivoclar Vivadent, Schaan, Liechtenstein)	*Powder* Barium aluminium silicate glass, ytterbium trifluoride, Isofiller, calcium barium alumino-fluorosilicate glass, calcium fluoro-silicate glass *Liquid* UDMA, DCP, aromatic aliphatic-UDMA, PEG-400 DMA, initiators, pigment
*Resin composite: Filtek™ Z350XT (FZ)* (3M ESPE, St. Paul, MN)	Bis-GMA, BIS-EMA, UDMA, TEGDMA, particles of silica and zirconia/silane, BHT, photoinitiator, pigments

*UDMA* urethane dimethacrylate, *DCP* triclodecan-dimethanol dimethacrylate, *Aromatic aliphatic-UDMA* tetramethyl-xylylen-diurethane dimethacrylate, *PEG-400 DMA* polyethylene glycol 400 dimethacrylate, *Bis-GMA* bisphenol A-glycidyl methacrylate, *BIS-EMA* bisphenol A-diglycidyl methacrylate ethoxylated, *TEGDMA* triethylene glycol dimethacrylate, *BHT* butylated hydroxytoluene

### Determination of biofilm pH

#### Bacterial preparation

*Streptococcus* (*S.*) *mutans* UA159 from frozen stocks (− 80 °C) was prepared as described by Wongpraparatana et al*.* with some modifications [11], cultured in a brain heart infusion (BHI) on an agar plate and incubated at 37 °C with 5% CO_2_ for 24 h. The isolated colonies were inoculated in BHI broth and incubated at 37 °C with 5% CO_2_ overnight. The overnight culture was adjusted to OD_600nm_ of 0.1 as measured by a spectrophotometer (Pharmacia LKB Biotechnology Inc, Uppsala, Sweden) and was further incubated until reach the log phase (OD_600nm_ = 0.5–0.6, approximately 3 h). The medium was changed from BHI broth to BHI broth supplemented with 5% sucrose for biofilm formation.

#### Biofilm formation and pH measurement

**Fig. 1:**
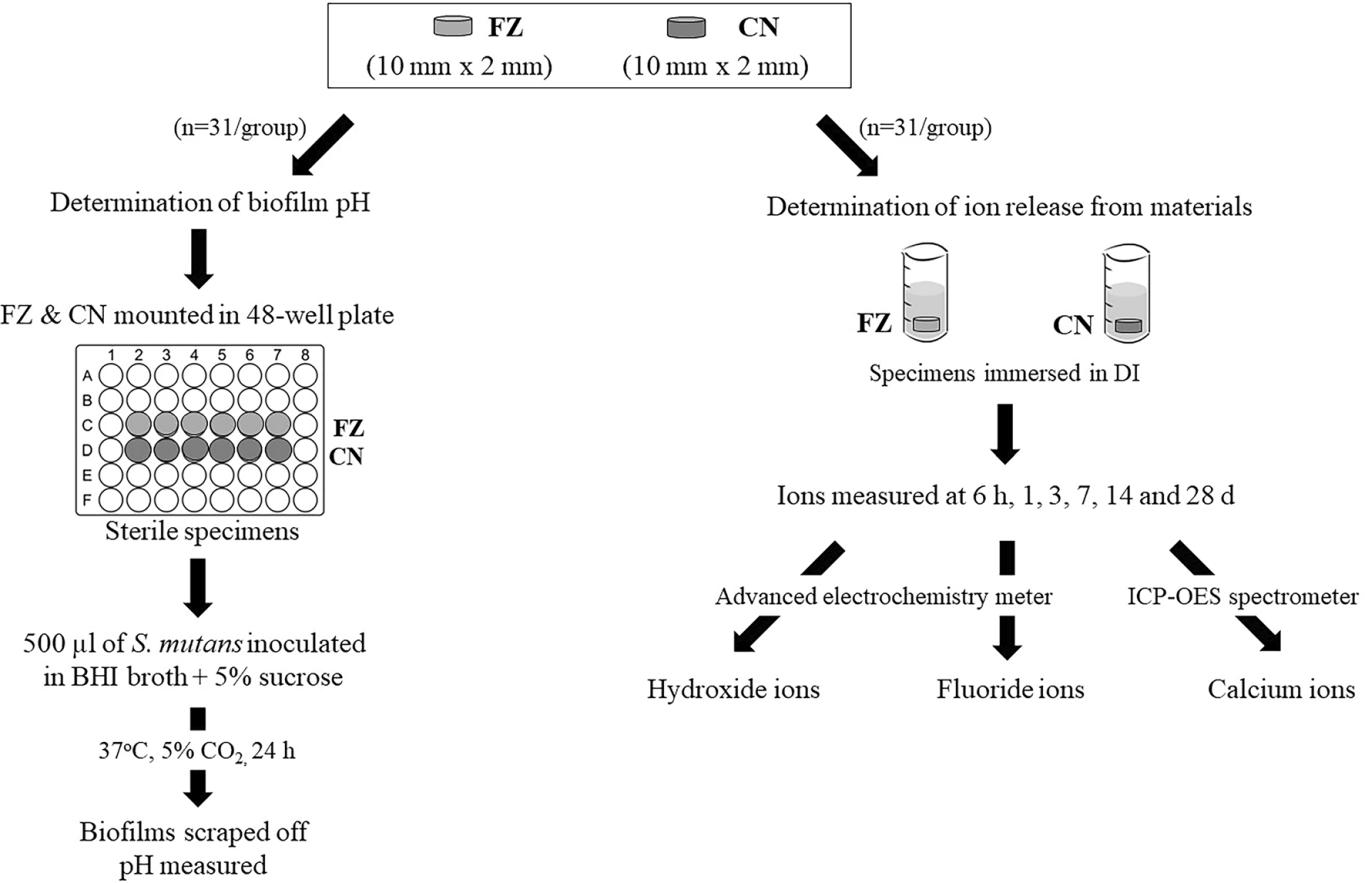
Flow diagram illustrating the determination of biofilm pH and ion release

The 31 FZ and CN specimens were embedded in a polyvinyl siloxane putty impression material in 48-well plates, with only the upper surface exposed. The specimens and plate were sterilized by plasma sterilization (STERRAD®100NX, ASP™, Irvine, CA). 500 µl of the prepared log-phase culture was added to each well and the plate incubated at 37 °C with 5% CO_2_ for 24 h to form a biofilm [11]. When the medium was removed after 24 h, the biofilm formed on the surface of the specimen was visible. The biofilms were then scraped off the specimens using a cell scraper and transferred to a pH meter (LAQUA pH-22, HORIBA, Kyoto, Japan), pre-calibrated using standard buffer pH values of 4.01, 7.00, and 10.01 [12], to measure the pH (Fig. 1).

### Determination of ion release

Thirty-one of each of FZ and CN specimens prepared as described above were individually immersed in 10 ml deionized-water (DI) and incubated at 37 °C [13]. Ions were measured after 6 h, 1, 3, 7, 14, and 28 days [10] (Fig. 1). The means and standard deviations (SD) of the amount of ions released were reported at each time point.

#### Hydroxide ions

The pH of the testing solutions and DI were measured using the advanced electrochemistry meter (Thermo Scientific™ Orion™ Versa Star™, Thermo Fisher Scientific, Waltham, MA). The molar (M) concentration of hydroxide ions ([OH^−^]) was calculated based on the equations [14]:

$${\text{pH}} + {\text{pOH}} = {14}$$

$${\text{pOH}} = \, - {\text{log}}[{\text{OH}}^{ - } ]$$

$$[{\text{OH}}^{ - } ] \, = {1}0^{{ - {\text{pOH}}}}$$

The concentration of hydroxide ion released by the materials was calculated using the formula:

$$[{\text{OH}}^{ - } ]{\text{released from materials}} = \, [{\text{OH}}^{ - } ]{\text{of testing solution}} - \, [{\text{OH}}^{ - } ]{\text{of DI}}$$

#### Fluoride ions [15, 16]

Fluoride ion release was determined using an advanced electrochemistry meter in ion-selective electrode (ISE) mode (Thermo Scientific™ Orion™ Versa Star™). Fluoride standard 100 ppm (Orion Calibration Standard 940,907) was diluted to three concentrations (0.1, 1.0, and 10 ppm) for ISE calibration. TISAB III solution (Orion Calibration Standard 940,911) was added to the test solution at a ratio of 1:10 by volume, and the probe placed into the solution to detect fluoride ion levels in ppm.

#### Calcium ions [17]

Calcium pure standard 1000 ppm solution (CAS Number 7440-70-2, Perkin Elmer®, Waltham, MA) was used to prepare 0.5, 1, 10, 50, and 100 ppm solutions and a calibration curve (CPS/ppm) made. Centrifuge tubes were immersed in 10% nitric acid solution for 24 h at room temperature and rinsed with DI. Ten ml of the test solution was filtered through Grade 1 filter paper, added to the centrifuge tube and the tube placed in the ICP-OES spectrometer (Optima^x^ 7300 DV, Perkin Elmer®). The concentration of calcium ions in counts per second (CPS) units were converted to ppm from the calibration curve.

### Determination of remineralization

#### Tooth specimen preparation

The sample size was calculated based on a previous study [18] using ANOVA: repeated measures, within-between interaction, a power of 80% and a significance level at 5%. The medium effect size of 0.25 was used for calculation. The study was approved by the Ethics Committee of Faculty of Dentistry, Chulalongkorn University (HREC-DCU 2020-040).

**Fig. 2:**
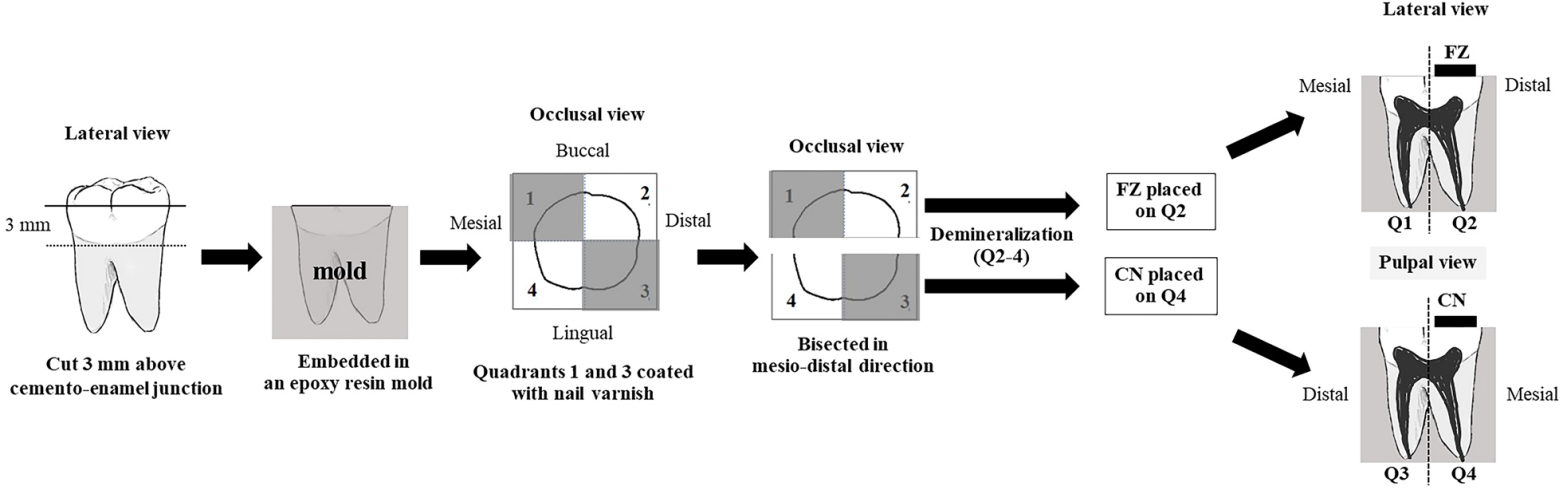
Specimen preparation. Q1 sound dentin; Q3 demineralized dentin control; Q2, and Q4 chemically demineralized dentin with FZ and CN placed on their respective surfaces

Human extracted molars were collected from dental clinics, cleaned using pumice-water slurry with rubber cup and an ultrasonic scaler, immersed in 0.1% thymol solution at 37 °C for 2 weeks and used within 2 months after extraction [19]. The fourteen upper and lower molars which were free of caries lesions, cracks, or restorations were included in this study. The teeth were sectioned horizontally approximately 3 mm above the cemento-enamel junction using a slow speed cutting machine (Isomet® 1000, Buehler, Lake Bluff, IL) with water coolant (Fig. 2). The occlusal section was discarded, and the radicular section embedded in epoxy resin in a 15 × 15 mm silicone mold, exposing only the cut surface (Fig. 2). The cut surface was divided into four quadrants, quadrants 1 and 3 coated with nail varnish, and the specimen sectioned in half mesiodistally along the border of the nail varnish. Quadrants 2, 3 and 4 were immersed in demineralization solution (50 mM acetate, 2.2 mM CaCl_2_·2H_2_O and 2.2 mM KH_2_PO_2_; pH = 4.6) for 96 h at 37 °C [20] and washed in DI for 30 s [21].

The FZ material was mixed as described above and bonded to quadrant 2 following the manufacturer’s instructions and using Clearfil™ SE Bond dentin bonding agent (Kuraray, Osaka, Japan). Briefly, the Primer was applied to the cut surface for 20 s and dried with a mild air flow. The Bond was applied, spread with a mild air flow and light-cured for 10 s (Elipar™ light; 3M, St Paul, MN). FZ was placed on the center of the cut dentin surface in a quarter circular metal mold, radius 7 mm, height 1 mm, and light-cured for 40 s. The CN material was mixed as described above, bonded to quadrant 4 using the adhesive and mold as described for FZ, and light-cured for 40 s. Specimens were immersed in DI at 37 °C for 30 days [22].

#### Microhardness measurement

**Fig. 3:**
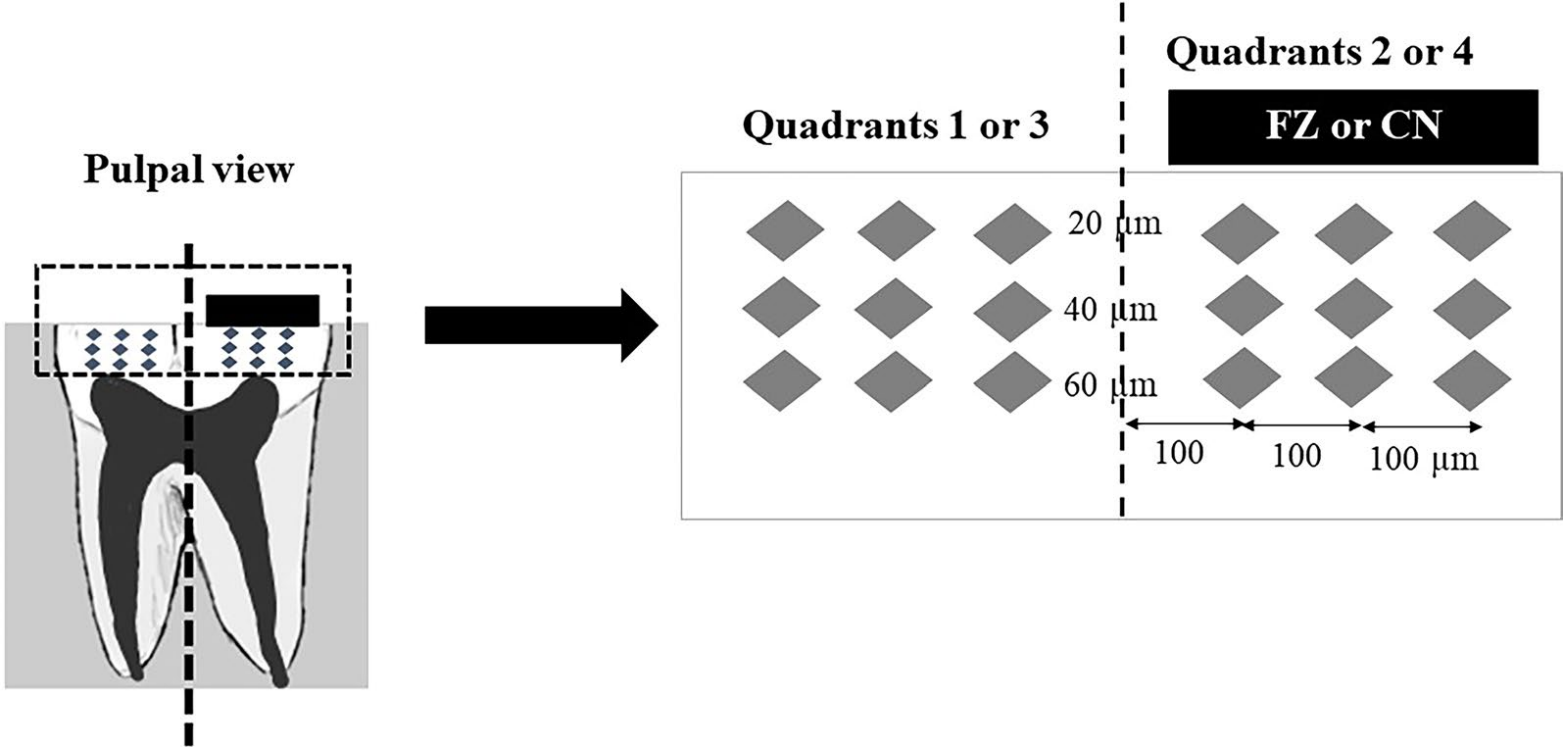
Microhardness measurement

Microhardness was evaluated using Knoop indenter with a load of 25 g for 15 s (FM-810, FUTURE-TECH, Kanagawa, Japan) [23]. Quadrants 1 and 3 were measured before and after demineralization respectively, and quadrants 2 and 4 measured 30 days after bonding the materials. Indentations were made at 20, 40 and 60 µm below the occlusal surface, and at the same depths, three spots 100 µm apart below the center of the materials (Fig. 3). The results were presented as the mean Knoop hardness number values (KHN) in the same row.

### Statistical analysis

The statistical analysis was done by SPSS version 28.0 software (SPSS Inc, Chicago, IL), using an independent t-test to compare the pH of biofilms between the FZ and CN groups. Ion concentrations released from FZ and CN specimens at each time point were compared using Mann–Whitney U test, and ion concentrations released from the same material at different time points were compared using repeated measure ANOVA.The repeated measures ANOVA with Bonferroni correction for multiple comparisons were used to compare the microhardness of dentin across groups at 20, 40, and 60 µm depth. Statistical significance level was set at $\mathrm{\alpha }$ = 0.05.

## Results

### pH of biofilm

**Fig. 4:**
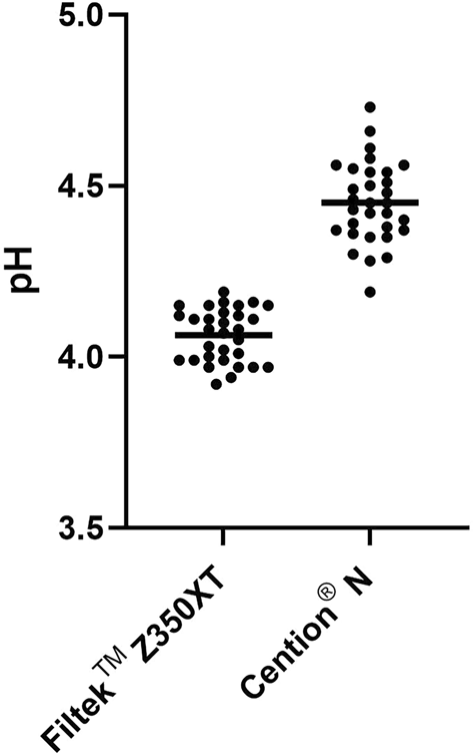
The pH of *S. mutans* biofilm formed on Filtek™ Z350XT (FZ) and Cention® N (CN) specimens. Each dot represents the pH of a specimen

The pH of the *S. mutans* biofilm on the CN restoration was 4.45, which was significantly higher than the pH of the biofilm on the FZ restoration (4.06; *p* < 0.001) (Fig. 4).

### Hydroxide ion release

**Fig. 5:**
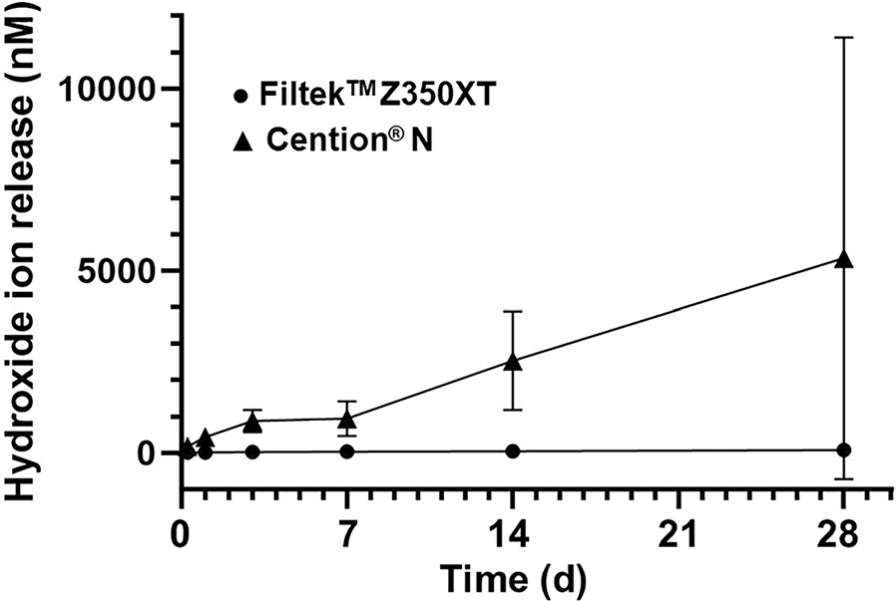
The mean of cumulative hydroxide ions released from Filtek™ Z350XT (FZ; Black filled circle) and Cention® N (CN; Black filled triangle) at six time points (6 h, 1, 3, 7, 14 and 28 days)

The cumulative hydroxide ions released from FZ and CN are shown in Fig. 5. The hydroxide ions of FZ were 6.4 ± 7.0, 8.8 ± 8.0, 22.1 ± 10.3, 33.5 ± 9.4, 44.6 ± 12.3, 74.7 ± 12.5 nM at 6 h, 1, 3, 7, 14 and 28 days, respectively, whereas those of CN were 176.4 ± 71.3, 438.3 ± 178.8, 881.8 ± 303.7, 1,500.4 ± 474.0, 2,533.8 ± 1,356.2, 8,154.9 ± 6,064.4 nM. Over the measured time intervals, the hydroxide ions produced by FZ increased by approximately 70 nM, whereas the hydroxide ions produced by CN increased by approximately 8000 nM and were significantly higher than FZ at each time point (*p* < 0.001). When comparing the number of ions at different time intervals within the group, a statistically significant difference (*p* < 0.001) was found in both groups.

### Fluoride ion release

**Fig. 6:**
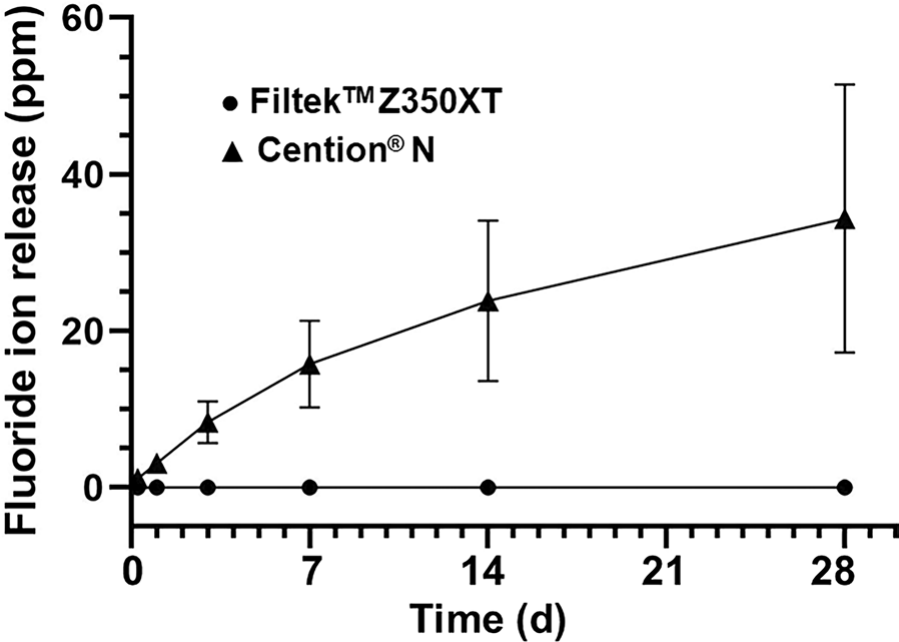
The means cumulative fluoride ions released from Filtek™ Z350XT (FZ; Black filled circle) and Cention® N (CN; Black filled triangle) at six time points (6 h, 1, 3, 7, 14 and 28 days)

The cumulative fluoride ions released from FZ and CN are shown in Fig. 6. The fluoride ions from FZ were 0.01 ± 0.00, 0.01 ± 0.01, 0.01 ± 0.01, 0.02 ± 0.01, 0.02 ± 0.01 and 0.03 ± 0.01 ppm at 6 h, 1, 3, 7, 14 and 28 d, respectively, whereas those from CN were 1.14 ± 0.33, 3.08 ± 0.72, 8.33 ± 2.68, 15.76 ± 5.55, 23.87 ± 10.26 and 34.33 ± 17.12 ppm, which were significantly higher than FZ at each time point (*p* < 0.001). Throughout the test period, CN continually released fluoride, accumulating a total of 34.3 ppm on day 28th. A statistically significant difference (*p* < 0.001) was detected in both groups when comparing the amount of ions at different time periods within the group.

### Calcium ion release

**Fig. 7:**
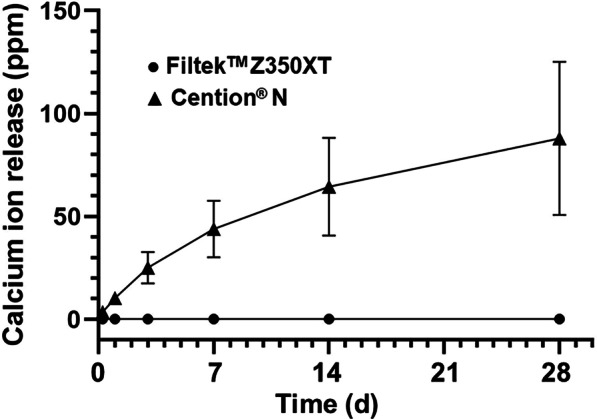
The mean cumulative calcium ions released from Filtek™ Z350XT (FZ; Black filled circle) and Cention® N (CN; Black filled triangle) at six time points (6 h, 1, 3, 7, 14 and 28 days)

The cumulative calcium ions released from FZ and CN was shown in Fig. 7. The calcium ions of FZ were 0.14 ± 0.13, 0.16 ± 0.13, 0.17 ± 0.14, 0.18 ± 0.15, 0.19 ± 0.14 and 0.22 ± 0.15 ppm at 6 h, 1, 3, 7, 14 and 28 d, respectively, whereas those of CN were 3.57 ± 1.12, 10.39 ± 2.69, 25.12 ± 7.60, 43.94 ± 13.71, 64.45 ± 23.71 and 87.88 ± 37.24 ppm. At each time point, the calcium ions released from CN were significantly higher than those released by FZ (*p* < 0.001); the total calcium ions from CN were 87.9 ppm after 28 days. At different time points, the calcium ion release from both groups was significantly different (p < 0.001).

### Knoop microhardness

**Fig. 8:**
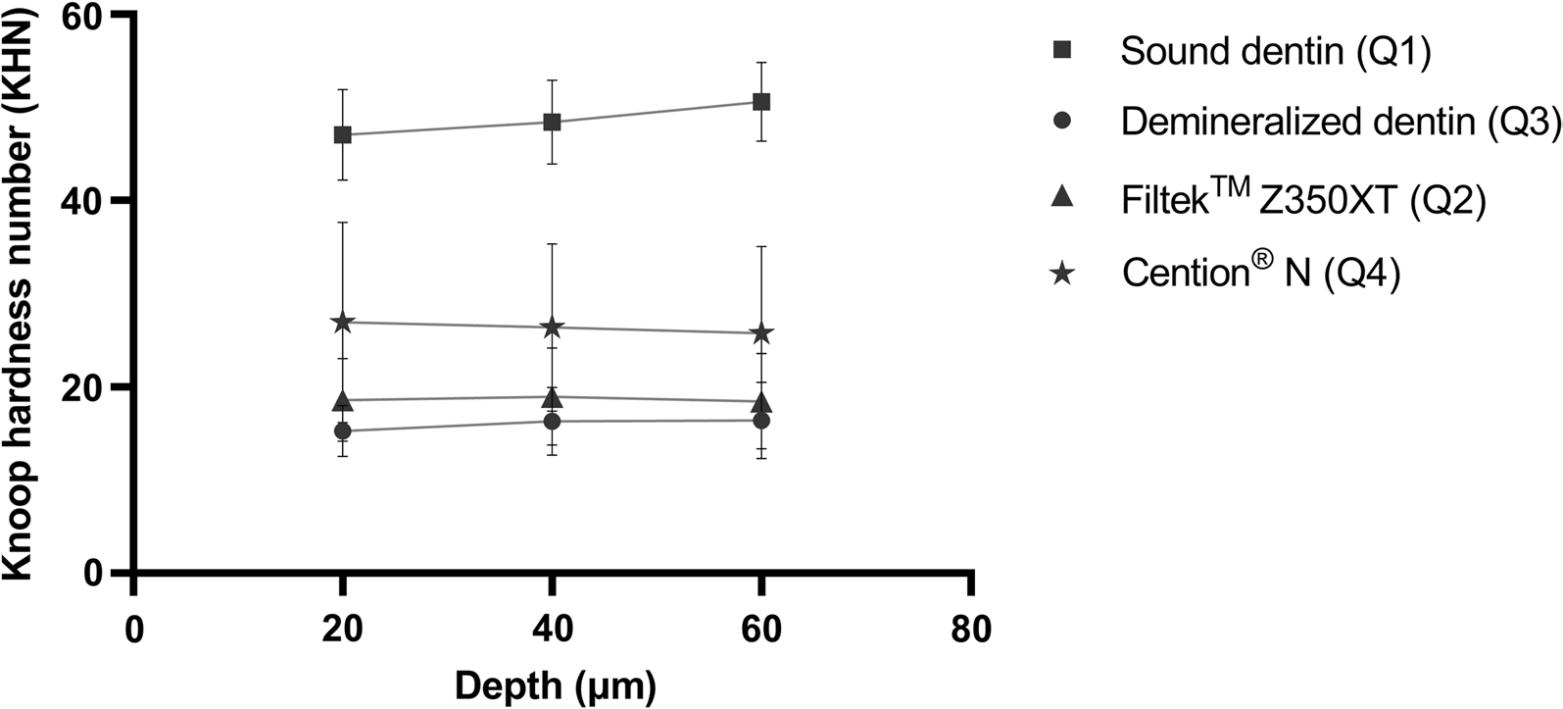
Mean value of Knoop hardness number (KHN) of dentin specimens at different depths

When the Knoop hardness number (KHN) was examined between depths of 20, 40, and 60 µm within each group, no difference was detected in all groups (*p* > 0.05) except that the KHN of sound dentin at 60 µm was significantly higher than at 20 and 40 µm (p = 0.03 and *p* = 0.02, respectively). However, when the groups were compared at the same depth, only specimens in the CN group had a significantly higher KHN than that in the demineralization group at all distances (*p* < 0.05), while the FN group specimens exhibited no difference (Fig. 8). Moreover, the KHN of the CN group specimens was significantly greater than that of the FZ group at 20 and 40 µm (*p* < 0.05, Fig. 8).

## Discussion

This laboratory study revealed that the CN had the capacity to increase the pH of a biofilm and the KHN of demineralized dentin more than did FZ. These findings were supported by the determination of ion release. The release of hydroxide, fluoride, and calcium ions was significantly higher from CN than from FZ at all time intervals (6 h, 1, 3, 7, 14 and 28 days), suggesting that the alkaline glass filler (calcium fluoro-silicate glass) is responsible for the substantial level of ions release from CN [6].

Over the period of 28 days, we found that CN continuously released hydroxide ions with a final concentration that was approximately 110 times greater than that from FZ. The release of hydroxide ion contributes to the regulation of the pH of the biofilm. This pH-regulatory property is useful in current caries management, which focuses on environmental control to accommodate commensal microorganisms, rather than eliminating pathogens [5]. As a result, hydroxide ion release may help preventing demineralization by increasing the pH of the acidic biofilm.

The results from present study showed that demineralized dentin subjacent to the CN restoration was higher in hardness compared to the control over a period of 30 d. Similarly, two previous studies using a nanoindenter and polarized light microscopy to detect the nanohardness and the demineralization inhibition effect respectively of CN at an enamel restoration margin, reported that the nanohardness of enamel adjacent to CN margin was not different from that when conventional glass-ionomer cement (GIC) was used [9, 24]. Furthermore, another study showed that the mean area of demineralization at the enamel and dentin adjacent to CN was significantly less than that adjacent to resin composite at 2 weeks [9]. In our study, we demonstrated that the release of calcium and fluoride ions from the CN material may result in the higher microhardness of CN at every depth compared to the control.

CN continuously released not only hydroxide ions, but also fluoride and calcium ions over a 28-day period, with final fluoride and calcium ion concentrations approximately 1300 and 400 times higher respectively than those from FZ. These findings were consistent with previous fluoride release studies, which showed that CN consistently released fluoride ions throughout the experimental periods of 21, 42, and 168 days, with a cumulative concentration of around 4, 7 and 12 ppm, respectively [10, 25, 26]. In the same way, Tiskaya et al*.* [10] demonstrated that CN can continue to release calcium ions up to 6 weeks, which corresponded with our findings.

Although the CN ion release profile found in our study was consistent with previous reports, the amount of released ions was not. The number of ions released from CN in the current study differed from the others because of various factors influencing the release such as specimen’s surface area**,** polymerization methods of materials (self-cured or light-cured), and storage media, including immersion time. Williams et al*.* [27] reported that the surface area of conventional GIC specimens affected fluoride ion release, with the greater the surface area, the greater the amount of fluoride ions released. Moreover, Tiskaya’s study [10] used 10 mm diameter and 1.2 mm thickness of CN specimen (surface area = 1.6 cm^2^) which had smaller surface area than ours (surface area = 2.2 cm^2^) and produced less fluoride and calcium ions. The number of ions released tends to increase in self-cured CN during immersion in an acidic media [10, 25]. It could be because light-cured polymerization results in a more tightly bound or less hydrophilic matrix, and a low pH solution induces more rapid degradation of glass filler and ion release when compared to a neutral solution [10, 25]. Several studies have shown that CN can release fluoride ions over long periods of time resulting in high fluoride accumulation [10, 25, 26, 28]. As a result, the amount of fluoride released is likely to be affected by the immersion time. CN has the ability to release fluoride ions, similar to conventional GIC and resin-modified glass-ionomer cement (RMGIC), nevertheless a difference in amount and pattern of fluoride ion release were found in a previous study [28]. Singh’s study [28] showed that conventional GIC and RMGIC released the highest fluoride ions in day 1 due to the burst effect, and the fluoride release gradually decreased over time. However, CN released the least fluoride ions in day 1 and significantly more fluoride than conventional GIC and RMGIC at all other time periods. From the literature, the polymerized resin matrix in RMGIC can limit water diffusion into the cement and impede the release of fluoride ions, which may be the same for CN [29, 30]. However, several studies, including ours, found that CN continuously released ions over the total experimental time, which may be due to the presence of 24.6% by weight alkaline filler in CN which is as much as one-third of all inorganic filler contained in CN [6]. Fluoride recharge is an important property of fluoride-releasing materials, and CN has been found to have this property as well as conventional GIC [31]. The number of porosities in a set material probably affects the quantity fluoride released both before and after recharge, resulting in GIC and RMGIC having more fluoride recharge ability than resin-based materials such as CN [32].

In a clinical context, when acids from cariogenic biofilm demineralize enamel, calcium and phosphate ions are released and accumulate in saliva. When the pH rises, minerals from highly saturated saliva return to the demineralized enamel, resulting in net mineral gain and hydroxyapatite structure repair. For this reason, adequate calcium and phosphate levels are critical for preventing mineral loss during low pH periods and encouraging mineral gain when the pH returns to normal [33]. When fluoride is present, the critical pH for calcium and phosphate ion solubilization decreases. Fluoride ions can integrate into a tooth by replacing hydroxide ions in hydroxyapatite to form fluorapatite or fluoride-enriched hydroxyapatite, which has a lower solubility than hydroxyapatite and calcium-deficient hydroxyapatite [34]. Because of this, the release of fluoride and calcium from CN may improve the inhibition of demineralization and the facilitation of remineralization. Several laboratory studies have demonstrated a higher potential of GIC and RMGIC than non-releasing fluoride materials to inhibit demineralization at a restoration margin [35–37]. Clinically, however the results are conflicting [38]. As mentioned above, CN can inhibit demineralization at the enamel and dentin adjacent to restorations in a similar way to conventional GIC, based on two laboratory studies [9, 24]. Until now, the evidence that CN can inhibit secondary caries or remineralize demineralized enamel and dentin is still weak without clinical support, thus it is necessary for further laboratory and in vivo studies to confirm the results.

## Conclusions

With the limitations of this laboratory study, we concluded that Cention® N, an alkasite restorative material, released hydroxide, fluoride, and calcium ions, which may result in an elevation of the pH of the *S. mutans* biofilm and an increase in the hardness of demineralized dentin. The results from this study suggested that Cention® N had the potential to reduce the incidence of secondary caries, which is a major cause of restorative treatment failure. However, because a variety of factors influence this ability, further research should be conducted in a clinical environment.

## Data Availability

The datasets are available from the corresponding author on reasonable request.

## Abbreviations

BHI: Brain heart infusion
CN: Cention® N
°C: Celsius
*S.**mutans*: *Streptococcus* *mutans*
DI: Deionized-water
FZ: Filtek™ Z350XT
g: Gram(s)
GIC: Glass ionomer cement
h: Hour(s)
KHN: Knoop hardness number
mm: Millimeter
µm: Micrometer
M: Molar
mM: Millimolar
mW/cm^2^: Milliwatt per square centimeter
µl: Microliter
nM: Nanomolar
ppm: Part per million
RMGIC: Resin-modified glass-ionomer cement
sec: Second(s)
